# The Anti-Venereal Campaign

**Published:** 1917-05-26

**Authors:** William Osler


### THE ANTI-VENEREAL CAMPAIGN.

### The Annual Oration Before the Medical Society

By SIR WILLIAM OSLER, BART., M.D., F.R.S.

At the annual meeting of the Medical Society, on Monday, May 14, under the presidency of Lieut.-Colonel D'Arcy Power, Sir William Osler, M.D., F.R.S., Regius Professor of Medicine at Oxford University, delivered the annual oration before a large audience on the subject "The Anti-Venereal Campaign."

### Sir William Osler's Address.

He said the past three years had seen the slaughter of man by man on a scale unseen before except perhaps in the lively narrative of the Book of Chronicles. But it was Apollo, not Mars, who slew more: disease was more fatal than war. More people died of plague in India in two years than had yet been killed on both sides in this great war. While nine soldiers died defending our country, twelve babies died at home. But the new knowledge had enabled the people to appreciate the relationship between bacilli and bullets. He considered that the story of the conquest of the great infections was the brightest chapter in the history of science. The vision, however, was darkened by two blots—cancer and venereal disease. A great awakening had taken place with regard to syphilis and gonorrhœa: among the infections they stand alone. Against all others man waged keen warfare. These two presented a remarkable combination of man and Nature in a successful propaganda against the health of the nation.

### Man and Nature Against National Health.

The report of Dr. Johnson, of the Local Government Board, and the Report of the Royal Commission on Venereal Disease, embraced the whole question. In this address he proposed to deal with only two points: the importance of the enemy, and the possibility of a successful campaign. In the returns of the Registrar-General for 1915 there was a remarkable absence of venereal disease as a cause of death in the review of vital statistics by Dr. G. H. C. Stephenson. Of eighteen causes of death there discussed all but three belonged to the infections; but there was no word about syphilis. Syphilis had been, and still remained, the despair of the statistician: trustworthy data about it were not forthcoming; even in death a stigma was associated with it. Deaths were accounted for under all conditions except under the caption of the disease itself. Only on two occasions was it alluded to—namely, on page 22, and then only casually. But at page 142 syphilis was stated to have been responsible for 1,885 deaths at all ages, and other venereal diseases for 61 deaths. Of the 1,885 deaths so caused 1,162 were in infants under a year old, 1,277 under five years. Syphilis was placed last in importance among ten causes of death, though it had been a very David among infections. They were given in the following order (omitting the chief killer of all, the diarrhœa of children): Tuberculosis, 54,000; pneumonia, 49,000; cancer, 40,000; measles, 16,000; influenza, 10,000; whooping-cough, 8,000; diphtheria, 6,000; scarlet fever, 2,000; cerebro-spinal fever, 2,000. For forty years physicians had consulted these reports as the sanitary index of the most sanitary country in the world. To have reduced the deaths from typhoid fever from 700 per million to 35 per million represented one of the decisive battles of this country. Even with hundreds of thousands of recruits of the most susceptible age, concentrated in camps, the death-rate from typhoid was the lowest in the country's history: our Army in France was enteric-free.

### The Great Race-Poisons.

Among the infections gonorrhœa and syphilis stood out as the great race-poisons. The gonococcus was not a great destroyer of life, but it was the great preventer of a full life; its cruel property was to sterilise a large proportion of its host. That was easily brought home by a study of gynæcological clinics and hospitals for diseases of women. A large number of the major operations were due to this cause. The percentage of sterility in women due to gonorrhœa was placed at 50, a large majority having acquired the disease innocently. Hence gonorrhœa cost the country annually thousands of lives.

### The only World-Wide Germ.

The spirochæte of syphilis was the only world-wide germ. Sir Arthur Newsholme had collected records of 100,000 still-births; how many of these were syphilitic was not known, but this disease was known to be the most common cause of abortion, and more than 25 per cent. of the still-born had been found infected. In 1915, of the 800,000 children born, 90,000 died in the first year. One-fifth of the latter number died in the first week, a fourth in the first month. Yet syphilis was not even mentioned as a cause, and in Dr. Stephenson's discussion of causes of infantile mortality, syphilis was only mentioned casually. Often the spirochæte lay dormant in the body, but it possessed the capacity to work evil which was not to be measured by years. "Venus of the Long Arm" she should be called, for after ten, twelve, twenty years the account was rendered and she wrung the uttermost farthing out of her victims. He had been in the habit of saying to his students, "Study one disease, study syphilis, study it thoroughly. All the others are added to you by the way; nearly all surgery, a great deal of medicine, and nearly all the specialties." It had been well said "Men do not die of the diseases that afflict them"; and that was illustrated by syphilis. Only 608 deaths among adults were given as due to syphilis; nothing could be more misleading. Serum reactions and recent knowledge had led to the public reading the chapter in the open.

### Syphilis as a Cause of Death.

Sir William proceeded to apportion the part probably played by syphilis in the causation of deaths attributed to particular organs, and in this way he arrived at the grand total of 60,000 as due to the spirochæte per annum, a result which moved syphilis from the tenth, in order of importance, to the top; it was an easy first among all the infections. In a series, one-third of the autopsies in adults showed the presence of spirochætes somewhere in the organism. Modern research led to the conclusion that there was an immense body of latent syphilis in the community, that a large number of the subjects of it had not been treated, and that in diseases of nerves, spine, and brain it was an enormous contributor. The President's (Mr. D'Arcy Power's) six-volume work on syphilis marked, with Sir Jonathan Hutchinson's report and the discovery by Ehrlich, a new view-point in regard to the disease. The Royal Commission report, the work of the National Council for Combating Venereal Disease (which provided accurate and enlightened information), had done a huge work towards furthering the campaign. The prevalence and seriousness of the position were shown by the last available figures for the British Army at home—namely, 71,000 cases of gonorrhœa, 21,000 cases of syphilis, 6,000 cases of soft chancre. The report for the Army in France was not available. In the Canadian Army there had been so many cases of syphilis and gonorrhœa that the public feeling of Canada had been aroused to boiling-point. The most sensible single piece of evidence before the Royal Commission was that of the Hon. Miss Brodrick, when she said we should deal with the disease as if it were smallpox or scarlet fever, quite apart from the moral side. This the Government had done.

### The Greatest Foe to Humanity.

He regarded tuberculosis as a more hopeful disease to fight than syphilis. Though tuberculosis had the same allies—poverty and drink—there was absent from it the complicating prostitution. No more hopeful legislation had ever been enacted than the establishment of these venereal clinics, by which the country would be well, but not fully, equipped for the battle. The people must realise that in this matter they were dealing with the greatest foe to humanity. He hoped that ere long the control of this matter would be directed from a Ministry of Health. Already the Commission had been able to do what the profession could not accomplish during long years: open the doors of the general hospitals to these victims, and the trustees had, at last, lined up on the side of the Good Samaritan. The profession welcomed the scheme from the educational side, as there would be easy opportunities of studying both disorders; and practitioners would bring their patients for treatment, for consultation, and for laboratory tests. He hoped that at each centre there would be lectures and demonstrations, such as at Liverpool. A sympathetic attitude on the part of general practitioners was essential to the work. He hoped the best men would be available for this campaign. The National Council could very well superintend it. It should not be left to the timid discretion of local authorities. All places of public resort, such as lavatories, should be utilised against advertising the venereal quack. People should be made to realise that it was a great communicable disease, and that two-thirds of its victims were innocent; then much of the ignorance and false sentiment in regard to the matter would be broken down. The confidence of the public must be gained and their feelings respected. He hoped there would be measures against the unauthorised treatment of the disease. Even the sympathy of the 5,000 men calling themselves herbalists should be enlisted; some of them were men of intelligence, and many were good botanists. The prime essential in a successful fight was to know where one's enemy was. The treatment must be thorough. In a series of cases 28 per cent. came only once.

### The Venereal Outlook.

To many the venereal situation looked dark and hopeless. It was not. For the first time the outlook was bright, even though there were 800,000 fresh cases every year. In the appeal to the people there was no reason why personal purity should not take first place. The reproach was not upon Christianity, but upon the frail vessels which held it.

### Sir Arthur Newsholme, C.B.

A cordial vote of thanks to the orator was proposed by Sir George Savage. Sir Arthur Newsholme, C.B., in seconding, said the Registrar-General's returns would more accurately represent the condition of things if students in medical schools were taught to properly fill up the certificates. There had been set up in Greater London twenty-two treatment centres for these diseases. During the first three months some 4,600 fresh cases of syphilis were treated. The number of cases of gonorrhœa was small, but in the next three months he knew they would be multiplied manyfold. He referred with satisfaction to the imminent passage of the Bill against treatment by quacks and the publication, public or private, of prescriptions for these diseases. The motion was supported by the President and carried with enthusiasm.

